# In Vivo Functional Analysis of the Human *NF2* Tumor Suppressor Gene in Drosophila

**DOI:** 10.1371/journal.pone.0090853

**Published:** 2014-03-04

**Authors:** Heather S. Gavilan, Rima M. Kulikauskas, David H. Gutmann, Richard G. Fehon

**Affiliations:** 1 Department of Molecular Genetics and Cell Biology, University of Chicago, Chicago, Illinois, United States of America; 2 Department of Biology, Duke University, Durham, North Carolina, United States of America; 3 Department of Neurology, Washington University School of Medicine, St. Louis, Missouri, United States of America; University of Massachusetts Medical School, United States of America

## Abstract

The proper control of tissue growth is essential during normal development and an important problem in human disease. Merlin, the product of the *Neurofibromatosis 2* tumor suppressor gene, has been extensively studied to understand its functions in growth control. Here we describe experiments in which we used Drosophila as an in vivo system to test the functions of the normal human *NF2* gene products and patient-derived mutant alleles. Although the predominant *NF2* gene isoform, isoform 1, could functionally replace the Drosophila *Merlin* gene, a second isoform with a distinct C-terminal tail could not. Immunofluorescence studies show that the two isoforms have distinct subcellular localizations when expressed in the polarized imaginal epithelium, and function in genetic rescue assays correlates with apical localization of the NF2 protein. Interestingly, we found that a patient-derived missense allele, *NF2^L64P^*, appears to be temperature sensitive. These studies highlight the utility of Drosophila for in vivo functional analysis of highly conserved human disease genes.

## Introduction

Neurofibromatosis 2 (NF2) is a dominantly inherited tumor predisposition syndrome characterized by the formation of schwannomas, meningiomas and other nervous system tumors [1]. In addition, mutations in the *NF2* gene have been associated with non-neuronal, sporadic cancers such as mesothelioma, suggesting that *NF2* has tumor suppressor functions in a variety of tissues. *NF2* encodes a membrane-associated cytoplasmic protein, called Merlin, which contains a Four-point-one, Ezrin-Radixin-Moesin (FERM) domain and is most closely related to the ERM (Ezrin-Radixin-Moesin) proteins [2]–[4]. FERM domains are believed to interact with the cytoplasmic tails of transmembrane proteins and bind other membrane-associated proteins [4], [5]. Thus Merlin likely acts at the cytoplasmic face of the plasma membrane [6], though some studies have suggested nuclear functions for Merlin as well [7].

Previous studies have identified a well-conserved *NF2* homologue, called *Merlin (Mer)*, in Drosophila [8]. Like its mammalian counterparts, Drosophila *Merlin* has growth suppressive functions as revealed by somatic mosaic analyses [9]. In addition, studies have demonstrated that Merlin protein is associated with endocytic processes, has functional interactions with Expanded, another FERM domain containing tumor suppressor, and works antagonistically to EGFR and other signaling pathways that function to promote proliferation in developing epithelia [10]–[12]. Recent work has also implicated Merlin and Expanded as upstream regulators of the Hippo pathway [13], [14], a well-conserved kinase-signaling cascade that is believed to control tissue growth during development and regeneration. Studies in mice indicate that Merlin also functions upstream of Hippo signaling in mammals [15], although the mechanisms by which Merlin regulates Hippo signaling might vary between different organisms. In addition, it is currently unclear whether all of Merlin's roles in tumor suppression are carried out through its ability to regulate Hippo signaling, or whether it might have some functions, such as regulation of EGFR accessibility at the cell surface and junctional organization, that are independent of the Hippo pathway [16], [17].

Given the similarities in structure and function between fly and mammalian genes and the utility of Drosophila genetics for studying gene function, we reasoned that Drosophila would be a valuable system in which to study the effects of splicing variants and disease associated mutations in human *NF2*. Here we describe functional analyses of an array of *NF2* forms using transgenic Drosophila as an in vivo ‘test tube’ for protein function. We show that although the predominant *NF2* isoform (Iso1) is able to rescue the lethality associated with Drosophila *Merlin* null mutations, a splice variant with a unique C-terminal tail does not. We find that like Drosophila *Merlin*, human *NF2* truncations still retain some, albeit weak, function. In addition, we provide evidence that a well-described severe patient derived mutation, *NF2^L64P^*, is a temperature sensitive allele that retains significant functionality at non-restrictive temperatures. Together these studies provide insights into *NF2* function and display the utility of Drosophila as an orthologous system for analysis of human disease genes.

## Materials and Methods

### *NF2* transgenes and genetic rescue experiments

Transgenes expressing the indicated *NF2* proteins were cloned with N-terminal Flag epitope tags in the pUASt transformation vector. P-element transformation [18] was used to generate multiple stable transgenic lines for each *NF2* allele tested. For genetic rescue tests, at least three independent autosomal insertion lines for each allele were crossed to female flies of the following genotype, *y w Mer^4^/FM7; T80-Gal4*, and for each of these lines, a sufficient number of offspring was scored to ensure that the predicted rescue class would number at least 40 flies. Percentage genetic rescue was calculated from the ratio of surviving *y w Mer^4^* hemizygous males over half of the total female offspring, thereby avoiding the semi-lethality associated with the *FM7* balancer chromosome in males. The *Mer^4^* chromosome was marked with *y* to allow us to distinguish between rescued males (with yellow body color) and males (normal body color) generated by patroclinous *X* chromosome inheritance due to meiotic non-disjunction in FM7 females.

### Immunofluorescence

Tissue dissection, fixation and antibody staining were performed as previously described [19]. Mouse anti-Flag (Sigma-Aldrich) was used at 1∶20,000 and guinea-pig anti-Merlin [8] was used at 1∶5000. Secondary antibodies (Jackson ImmunoResearch) were diluted 1∶1000 and tissues were mounted in Prolong Antifade (Molecular Probes). Confocal images were taken on either a Zeiss LSM 410 or LSM 510 microscope using Zeiss acquisition software (Carl Zeiss MicroImaging).

### Analysis of wing phenotypes

The effect of ectopic expression of the *NF2* isoforms under the control of the *apterous-Gal4* driver was examined in adult wings. Wings were prepared by incubating flies in 70% EtOH, rinsing in water, and then mounting the dissected wings in Aquamount (BDH Laboratory Supplies). Wings were imaged under brightfield optics on a Zeiss Axioplan 2ie compound microscope and recorded using a Spot digital camera.

### Quantitative PCR

Total RNA was prepared from 20 wing imaginal discs from larvae of the following genotypes – *ApGal4/+* (control), *ApGal4/P[hNF2^Iso1^]; P[hNF2^Iso1^]/+, ApGal4/+; (2X)P[hNF2^Iso2^]/+* - using Trizol (Invitrogen). cDNA was produced using the Taqman kit (ABI) and quantitative PCR performed using the LightCycler FastStart SYBR green system (Roche).

## Results

### Genetic rescue of Merlin mutations by NF2 alleles

**Figure 1:**
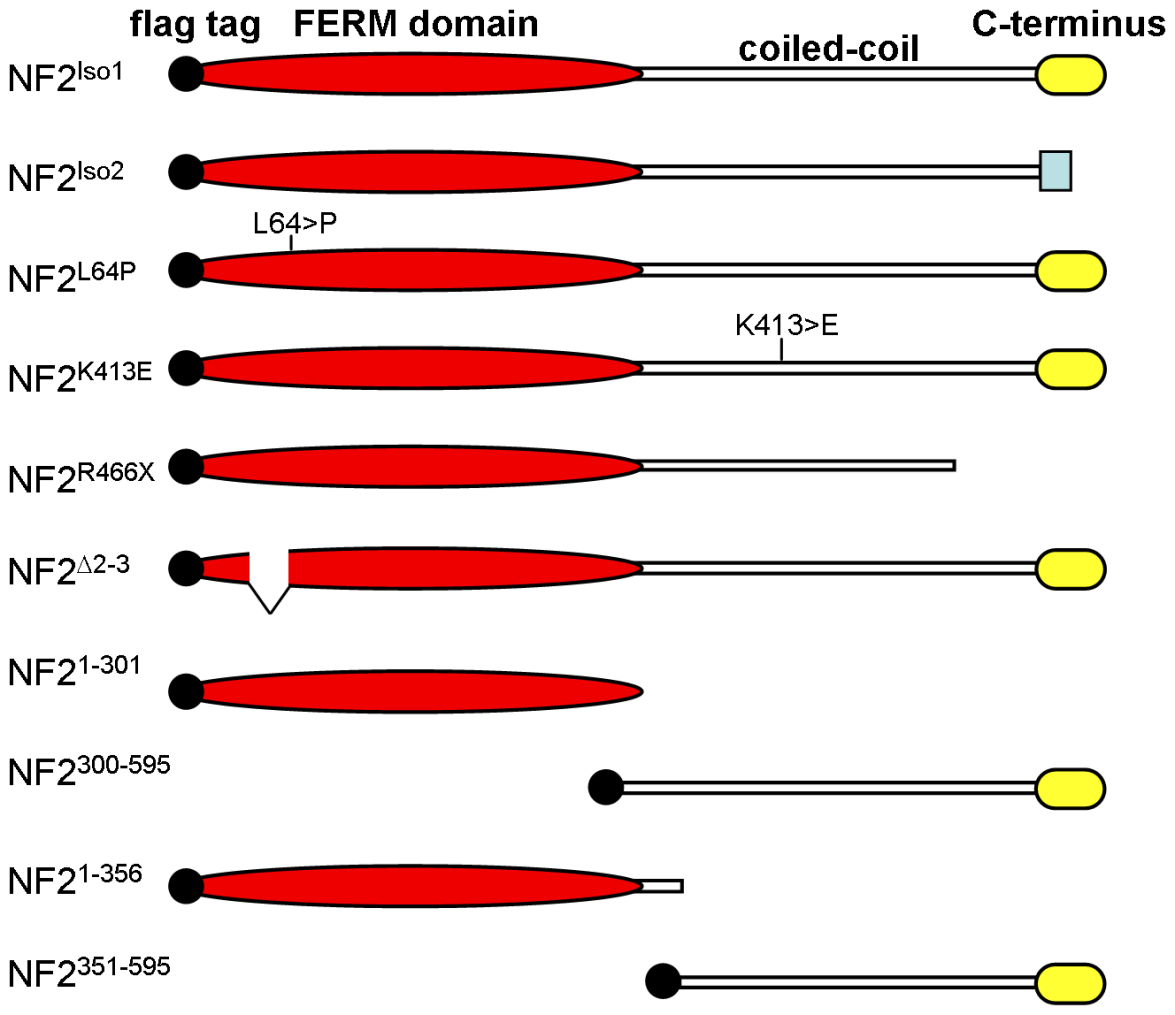
Diagrams of hNF2 proteins used in this study. The known protein domains, including the FERM domain, the coiled-coil domain, and the C-terminal FERM binding domain, are indicated as shown. All constructs are N-terminally tagged with the FLAG epitope. Details of the mutant alleles are provided in the text.

**Figure 2:**
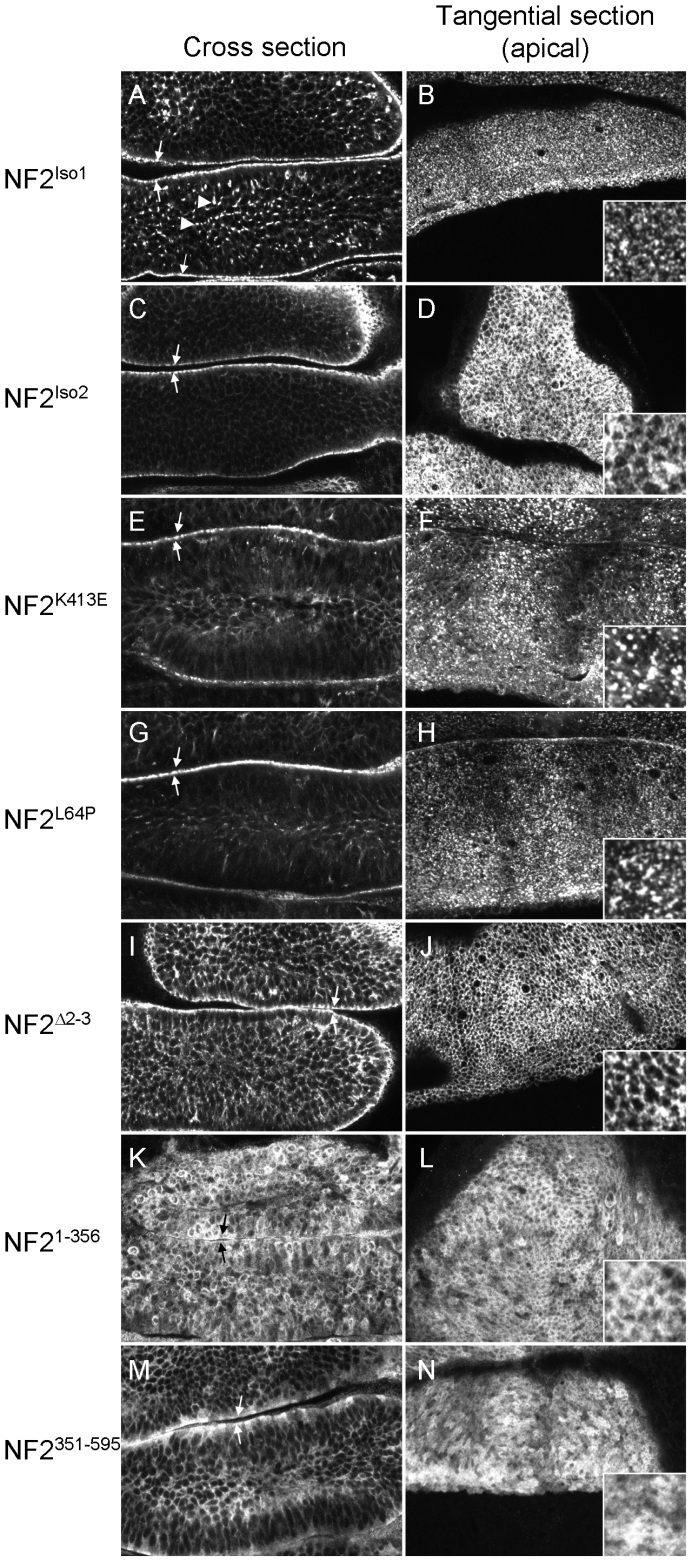
Mutations in *hNF2* alter the subcellular localization of the Merlin protein expressed in Drosophila. Confocal optical sections of wing imaginal epithelia, either cutting deeply into the epithelium (‘Cross section’) or tangentially through the apical surface of the epithelium, are presented for each allele or isoform. A–B) NF2 isoform 1 is primarily localized to the apical membrane (arrows) of these polarized epithelial cells, and adopts a punctate appearance at the apical cortex that is not clearly associated with cell boundaries (see inset at right). In addition, some punctate staining is observed basally (arrowheads in A). C–D) In contrast isoform 2, while still primarily apical (arrows, C), appears associated with cell boundaries (outlining the periphery of the apical ends of cells in D) and does not form punctae. The localizations of the K413E (E–F) and L64P (G–H) point mutations appear similar to that of isoform 1. The Δ2-3 deletion allele (I-J) appears localized to the lateral cell cortex, somewhat similar to isoform 2, but less strongly apically localized (arrows mark apical; also note staining throughout the basolateral domain in I). NF2^1-356^ (K–L) is substantially cytoplasmic and fails to accumulate apically while NF2^351-595^ (M–N) shows some apical enhancement (arrows in M). Both truncations display substantial cytoplasmic localizations, as indicated by the tangential views (compare L and N to J).

We first asked if expression of the two known isoforms, *NF2^Iso1^* and *NF2^Iso2^* [20], could functionally replace the endogenous *Merlin* gene in a transgenic rescue assay (Fig. 1). Isoform 1 has homology with Drosophila Merlin and the ERM proteins along its entire length. In contrast, isoform 2, which is formed by the insertion of an exon near the 3′ end of the coding sequence (exon 16) that replaces the C-terminal 16 amino acids of isoform 1 with a novel stretch of 11 amino acids, has a unique C-terminal tail. All transgenic constructs were Flag epitope tagged at the amino terminus (Fig. 1; Materials and Methods). Ability to rescue was tested using ubiquitous expression via the *Gal4*/*UAS* system in animals hemizygous for the *Mer^4^* null allele, which severely truncates the Merlin protein [9]. In all rescue experiments, at least three independent *NF2* transgenic lines of each construct were tested to control for expression differences due to insertion site. Also, immunostaining using anti-Flag antibody (Fig. 2) and immunoblot analysis (data not shown) were used to confirm expression of all *NF2* forms.

As shown in Table 1, expression of a transgene expressing *NF2^Iso1^* using the ubiquitously expressed T80 Gal4 driver [21] substantially rescued the lethality of the null *Mer^4^* allele, while *NF2^Iso2^* provided very poor rescue. Interestingly, the rescue provided by *NF2^Iso1^* improved slightly with increased genetic dose, while *NF2^Iso2^* rescue seemed to decrease with increasing dosage. This behavior would be expected for an allele with dominant negative effects on the low levels of maternally encoded Merlin found in *Mer^4^* zygotes, and clearly indicates that isoform 2 has significant functional differences from isoform 1 in this assay. This conclusion is consistent with a recent study demonstrating an isoform 2-specific neural function in the mouse [22].

**Table 1:** Genetic rescue by hNF2 alleles.

Allele	% rescue (1 dose^1^)	%rescue (2 doses^2^)
Isoform 1	59±7	72
Isoform 2	8±4	0
L64P	73±6	71
K413E	36±10	62
R466X	0±0	11
Δ2–3	0±0	0
1–301	0±0	0
300–595	2±1	0
1–356	0.2±0.2	0
351–595	0±0	0

1 Average of three or more independent lines.

2 Using two of the insertions tested in the one-dose experiments.

Studies of NF2 patient families have identified a number of point mutations in the *NF2* gene. Most are nonsense mutations, but there are a small number of known disease-causing missense mutations that we reasoned could be used to provide insights into structure/function relationships in the Merlin protein [23]. Given that *NF2^Iso1^* provides significant rescue of *Mer* null mutations in flies, we generated transgenes carrying these disease-associated *NF2* alleles and tested their function in the rescue assay (Fig. 1; Table 1). Interestingly, the *NF2^L64P^* missense mutation, which has been proposed to be strongly inactivating [24], provided complete genetic rescue in our assay. Another disease-associated mutation, *NF2^K413E^*, showed reduced rescue ability in a dose sensitive fashion, suggesting that it is hypomorphic for *Merlin* function.

To further analyze structure/function relationships in *NF2*, we generated deletion transgenes and tested their ability to rescue *Merlin* lethality. Multiple C-terminal deletions were tested - deletions of the C-terminal half of Merlin (*NF2^1-301^*, *NF2^1-356^*), and a disease associated nonsense mutation (*NF2^R466X^*). While *NF2^1-301^* and a slightly larger form *NF2^1-356^* failed to provide rescue at any dose, the *NF2^R466X^* allele, which encodes more of the C-terminal domain, rescued lethality moderately when expressed at the higher level afforded by two copies of the transgene (Table 1). This observation suggests that this allele is hypomorphic and is consistent with previous studies of Drosophila Merlin that indicate that the C-terminal region of the protein contains non-essential regulatory sequences [9], [14]. In contrast, expression of the C-terminal domain alone (*NF2^300-595^* and *NF2^351-595^*) was not sufficient to provide rescue. Expression of a form that lacks the coding sequences provided by exons 2 and 3 (*NF2* ^Δ*2-3*^), which has been proposed to have dominant-negative activity [25], similarly failed to provide any genetic rescue.

### Subcellular localizations of NF2 proteins

To learn more about the functions of these *NF2* transgenes, we examined the subcellular localization of their products when expressed in the larval imaginal discs. These structures, which produce adult appendages such as wings and legs during metamorphosis, consist of a single layered columnar epithelium that displays a highly structured apical/basal epithelial polarity. Merlin functions to control proliferation in these tissues, and the Merlin protein is primarily localized to the apical membrane and junctional domain [8], [9]. NF2^Iso1^ expressed in the wing imaginal epithelium under the *apterous Gal4* (*apGal4*) driver was highly concentrated at the apical domain, in a distinctly punctate pattern (Fig. 2A–B). In addition this protein localized to punctate structures below the apical surface, consistent with previous studies indicating that Merlin is associated with endocytic compartments [8], [26]. NF2^Iso2^ was also apical, but lacked the punctate appearance and instead was associated with cell boundaries at the apical ends of epithelial cells (Fig. 2C–D). As observed in the genetic rescue experiments (Table 1), this result suggests significant functional differences between these two NF2 isoforms. Consistent with this, the two other proteins that provided significant rescue, NF2^L64P^ and NF2^K413E^, had subcellular distributions that were indistinguishable from that of NF2^Iso1^ (Fig. 2E–H). In contrast, NF2^Δ2-3^ was primarily associated with the lateral cell cortex (Fig. 2 I–J) and the N- and C-terminal truncations were more cytoplasmic in localization (Fig. 2 K–N). Similar localizations were observed when the each transgene was present in either one or two doses, indicating that differences in expression level do not affect subcellular localization.

**Figure 3:**
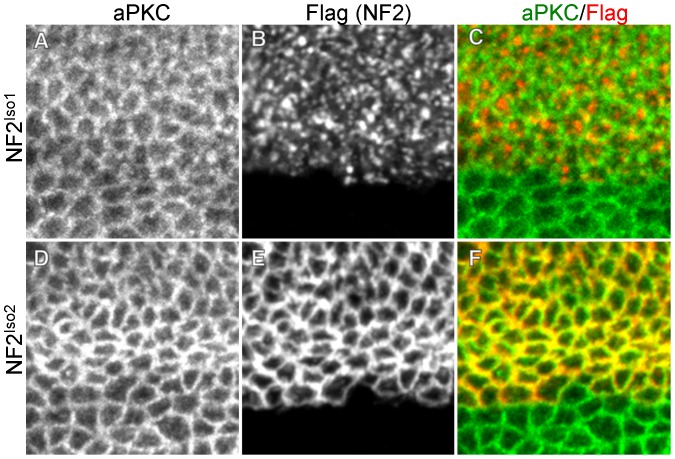
NF2 isoforms 1 and 2 display distinct localizations within the apical domain. Wing imaginal discs expressing Flag-tagged NF2^Iso1^ (A–C) or NF2^Iso2^ (D–F) under the ApGal4 driver. Images are taken along the dorsal-ventral boundary to include the expression boundary of ApGal4, thereby demonstrating the specificity of anti-Flag staining in these tissues. Comparison to aPKC, a marker for the marginal zone in these cells (A,D), demonstrates that NF2^Iso1^ (B–C) is expressed in the apical cell cortex but is not closely associated with the marginal zone. In contrast, NF2^Iso2^ (E–F) is highly concentrated in the marginal zone together with aPKC.

To better characterize the localizations of isoforms 1 and 2, we co-stained for these proteins and atypical protein kinase C (aPKC) in the wing imaginal epithelium. Previous studies have shown that aPKC is concentrated in the marginal zone, a region of the lateral cell membrane just apical to the adherens junction, in these cells [27], [28]. As expected for a marginal zone component, aPKC localized junctionally in the apical region of wing imaginal disc cells (Fig. 3A,D). Strikingly, while NF2^Iso1^ was also predominantly localized apically, it did not show the same ‘fishnet’ staining pattern observed for aPKC, and did not strongly colocalize with aPKC (Fig. 3A–C). In contrast, NF2^Iso2^ staining was quite similar to aPKC, and strongly colocalized with aPKC (Fig. 3D–F). These staining patterns indicate that NF2^Iso1^ is distributed in a punctate, non-junctional pattern across the apical cell cortex, while NF2^Iso2^ localizes primarily in the apical junctional region. Thus, there was a correlation between punctate, apical localization and ability to provide genetic rescue, suggesting that this localization is important for Merlin function.

### Effect of NF2 expression on endogenous Merlin

**Figure 4:**
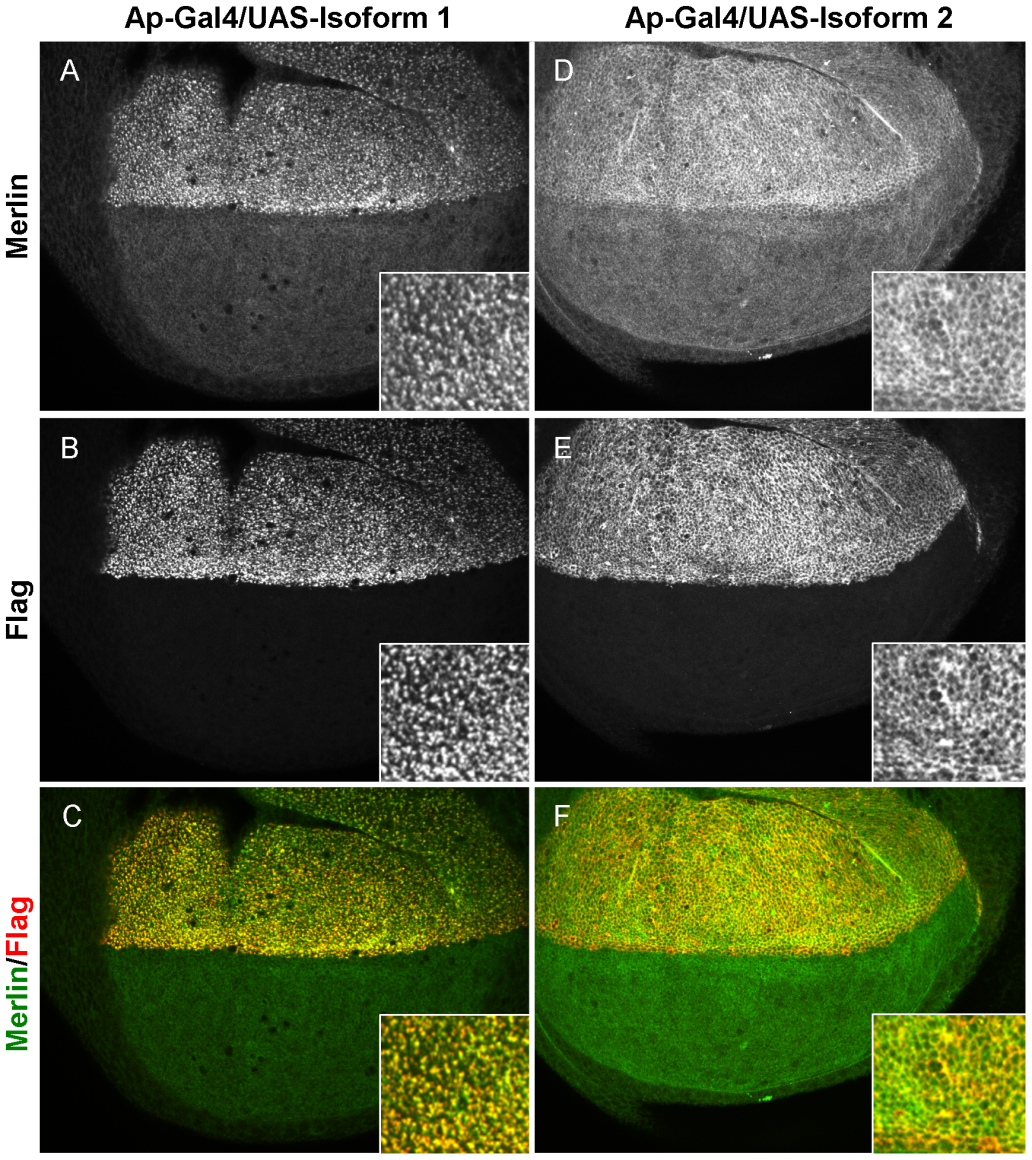
Expression of hNF2 isoforms affects expression and localization of endogenous Drosophila Merlin. Larval wing imaginal discs are shown stained for hNF2 (A, C) or Drosophila Merlin (B, D) proteins. hNF2 expression is driven specifically in the dorsal half of the wing epithelium by the *apterous*-Gal4 driver. Images are projections of a series of tangential confocal optical sections, showing protein primarily localized to the apical surface of the epithelium. Expression of isoform 1 (A–B) results in increased Merlin protein levels and redistribution into punctate structures at the apical surface. In contrast, expression of isoform 2, while also causing increased expression of Merlin, does not cause redistribution away from the junctional region. Insets at lower right of each panel show higher magnification views of subcellular localization.

Previous studies of Merlin and the closely related ERM proteins have demonstrated that these proteins display both homotypic and heterotypic intermolecular interactions in vitro [29], [30]. This led us to ask if *NF2* expression resulted in alterations in expression or localization of endogenous Drosophila Merlin in these tissues. Expressing *NF2* in the dorsal half of the epithelium under the *apGal4* driver, allowed us to make side-by-side comparisons of Merlin in cells that did or did not express *NF2*. Control experiments, in which isoform 1 was expressed in a *Mer^4^* null mutant background, demonstrated that our anti-Drosophila Merlin antiserum (raised against the less conserved carboxyl terminal half of the protein [8]) specifically recognized the fly protein and did not cross react with human Merlin (data not shown). We then used this antibody to compare Merlin localization in cells that express NF2 ectopically to Merlin localization in normal epithelial cells. Expression of NF2^Iso1^, which strongly rescued null *Mer* mutations (Table 1), affected both the apparent amount and localization of endogenous Merlin. As shown in Fig. 4, endogenous Merlin staining was much more abundant in cells that express NF2^Iso1^. In addition, although Drosophila Merlin is normally primarily junctional in imaginal epithelial cells [8], it appeared localized to the apical cell cortex with NF2^Iso1^ in cells expressing both proteins. This degree of redistribution and colocalization suggests that Drosophila Merlin and NF2^Iso1^ interact when expressed in these tissues. Imaginal discs expressing NF2^Iso2^ also showed increased endogenous Merlin staining in the dorsal compartment, though in this case Merlin was predominantly associated with cell boundaries in the apical region of cells, mirroring the difference we observed between NF2^Iso1^ and NF2^Iso2^. Consistent with the idea that the effects of *NF2* expression on endogenous Merlin were posttranscriptional, quantitative PCR analysis failed to show any detectable effect on Merlin transcription in wing imaginal discs expressing either *NF2* isoform under the *apGal4* driver (Table 2).

**Table 2:** qPCR analysis of effect of hNF2 expression on endogenous Merlin expression.

Experimental replicate	net ΔMerlin^1^	
	*ApGal4-2x[NF2^Iso1^]*	*ApGal4-2x[NF2^Iso2^]*
1	1.39	0.93
2	0.84	0.65
3	1.01	0.97
Mean	1.08	0.85
SD	0.28	0.18

1 Fold expression change compared to control genotype, *ApGal4/+.*

### Dominant negative effects of *NF2^Iso2^*

**Figure 5:**
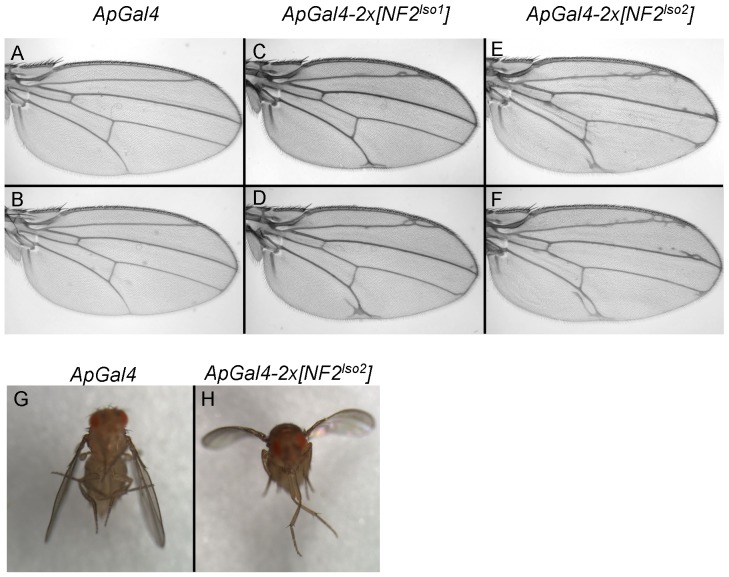
Ectopic expression of hNF2 isoforms 1 and 2 has different effects on Drosophila wing development. Two transgenic copies of each isoform were expressed in the dorsal half of the developing wing blade under the control of the *apterous*-Gal4 driver. The apterous-Gal4 driver itself has little or no effect on wing development (A–B), while expression of isoform 1causes some ectopic vein formation, particularly along the second and fifth wing veins (C–D). Expression of isoform 2 (E–F) causes more severe ectopic vein formation that includes veins 2, 3 and 5. In addition, expression of this isoform causes a downward cupping of the wing blade (data not shown).

To further test the functions of these *NF2* transgenes in developing Drosophila tissues, we examined their adult phenotypes when expressed under the control of the *apGal4* driver, which is expressed only in the dorsal half of the wing imaginal disc. Our previous work with Drosophila Merlin showed that overexpression of a *Mer^+^* transgene in the wing has no phenotype [9]. However, overexpression of *NF2^Iso1^* did show some dominant phenotypes in the wing, most typically ectopic vein material along veins II and V (Fig. 5C–D). Expression of *NF2^Iso2^* produced qualitatively similar ectopic wing vein material (Fig. 5E–F). However unlike *NF2^Iso1^*, we observed a marked downward ‘cupping’ of the wing blade when *NF2^Iso2^* was expressed in the dorsal half of the wing (Fig. 5G–H). This phenotype is characteristic of overproliferation in the dorsal wing compartment, and has been seen previously with a dominant-negative *Merlin* transgene expressed by the *apGal4* driver [9]. This result suggests that *NF2^Iso2^* dominantly interferes with endogenous *Merlin* function, consistent with the results from rescue assays (Table 1). In support of this notion, the severity of *NF2^Iso2^* induced wing vein phenotypes was enhanced in *Mer^4^/Mer^+^* females in comparison to *Mer^+^/Mer^+^* flies (data not shown).

### The *NF2^L64P^* allele is temperature sensitive

**Figure 6:**
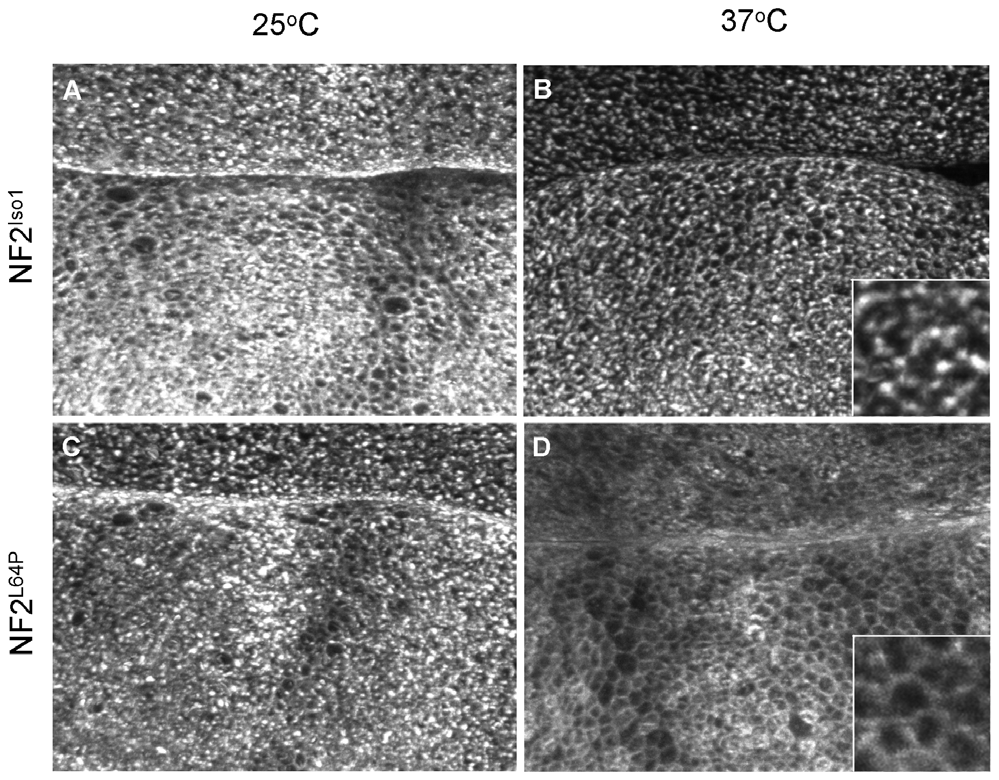
The *NF2^L64P^* mutation affects subcellular localization in a temperature sensitive fashion. Subcellular localizations of expressed NF2 isoform 1 (A–B) and the L64P mutation (C–D) are compared in wing imaginal discs kept at 25°C (A,C) or 37°C (B,D) for two hours prior to dissection. While the L64P mutant protein shows normal apical, punctate localization at 25°C (C), it is much more cytoplasmic and evenly distributed at 37°C (D). In contrast, the higher temperature has no apparent effect on subcellular distribution of the wild type isoform 1 protein, which was apical and punctate at both temperatures (A–B). Insets in B and D show higher magnification views of representative areas.

It was surprising to find that the *NF2^L64P^* mutation, a patient-derived mutation [24], appeared to function equivalently to wild-type *NF2* in the genetic rescue assay (Table 1). This result is particularly unexpected in light of studies that have reported significant differences between wild-type and NF2^L64P^ mutant protein in mammalian cell-based functional assays due to misfolding of the protein [23], [24]. For these reasons, we speculated that *NF2^L64P^* might be temperature sensitive and that the differences between observed *NF2^L64P^* function in mammalian and Drosophila cells relate to the difference in temperature at which these organisms normally function – our in vivo Drosophila assays are conducted at 25°C while mammalian cells are typically cultured at 37°C. Because flies cannot survive when raised at 37°C, to test this possibility we expressed the *NF2^L64P^* transgene in larval wing imaginal epithelial cells, pulsed the animals at 37°C for two hours, and then used immunofluorescence staining to determine the subcellular localization of the expressed protein. Side by side comparison with either NF2^L64P^ expressing animals maintained at 25°C, or with animals expressing NF2^Iso1^ similarly pulsed at 37°C, showed an obvious mislocalization of the mutant protein at 37°C (Fig. 6). In particular, the punctate apical staining that is characteristic of NF2^Iso1^ was disrupted when animals expressing the NF2^L64P^ protein were maintained at 37°C for two hours, and instead the mutant protein appeared largely cytoplasmic as observed previously in mammalian cells [31]. Given the correlation between apical, punctate localization and functionality in the rescue assay described above, these results strongly suggest that NF2^L64P^ is functional at 25°C but not at 37°C, and therefore that this allele is temperature sensitive.

## Discussion

Taken together, the results presented here reveal several aspects of *NF2/Merlin* function relevant to its role in tumor suppression and the evolutionary conservation of its functions. Our in vivo studies in the fly reveal clear differences in genetic function and subcellular localization in epithelial tissues between *NF2* isoforms 1 and 2, which differ only in the C-terminal 16 amino acid residues. This is particularly interesting in light of a recent study showing that genetically deleting either isoform alone does not result in lethality or increased tumor formation in mice [22] indicating substantial functional redundancy between the two isoforms. In contrast, in flies isoform 2 is unable to compensate for loss of endogenous Merlin, and appears to dominantly interfere with endogenous Merlin activity in restricting proliferation in the wing epithelium. Thus, our results suggest that isoform 2 could, at least in some contexts, antagonize isoform 1 function in proliferating tissues. This activity could be regulated through interactions with other proteins, consistent with previous studies that have identified isoform-specific NF2 binding partners [22], [32], [33]. Indeed, Schulz et al. [22] demonstrate that isoform 2, but not isoform 1, forms a complex with RhoGDI and promotes RhoA activation in neurons. This function is also unlikely to be recapitulated in fly cells because the phenotypes in the wing we observed from ectopic expression of isoform 2 (Fig. 4) do not resemble those previously described for increased Rho1 function [34].

An interesting implication of these studies relates to the question of how well Merlin function has been conserved evolutionarily. At the protein level, Merlin is 55% identical between flies and mammals [8], and as we have shown here, *NF2* can genetically rescue null *Merlin* mutations in flies. Recent work in flies has strongly implicated Merlin as an upstream regulator of the Hippo pathway, and suggests that all Merlin functions in growth control are mediated through its effects on Hippo signaling. In mammalian cells it is less clear whether all Merlin functions are directly related to the Hippo pathway [17], though it seems that at least some of them are [15]. Whether some of this functional diversity is due to isoform 2, which is unique to mammals, remains to be seen, but its unique properties are consistent with this possibility.

Strikingly, the *NF2^L64P^* allele, which is reported to be a strongly inactivating allele in humans [24], displays essentially wild-type genetic rescue and subcellular localization in Drosophila reared at 25°C, suggesting that this missense mutation is temperature sensitive. Consistent with this, we found that while normal at 25°C, localization of NF2^L64P^ was dramatically altered at 37°C. Similar behavior has been seen previously for other disease-causing mutations [35], [36]. If correct, then our model for NF2^L64P^ suggests that approaches based on correcting protein folding defects might be therapeutically effective for treating this disease causing mutation in the *NF2* gene. A second disease related allele we examined, *NF2^K413E^*, appeared hypomorphic in our in vivo assays, while a previous study in mammalian cells indicated that it severely affects *NF2* function [24]. This allele could also be temperature sensitive, though it is also possible that this functional distinction simply reflects the differences in the assays used in the two studies.

Our studies suggest a strong relationship between subcellular localization in polarized epithelial cells and proper *NF2* protein function. All *NF2* proteins providing genetic rescue in flies, NF2^Iso1^, NF2^K413E^, and NF2^L64P^, localize to the apical cell cortex in the imaginal epithelium. Interestingly, we observed that two dominant negative *NF2* proteins, NF2^Iso2^ and NF2^Δ2-3^ [25], are predominantly localized to the lateral plasma membrane, as is true for the dominant negative delta-blue-box allele of fly Merlin [9]. Based on these results, and previous studies in mammals that indicate that Merlin functions to regulate both receptor availability [11], [37] and Hippo pathway activity [38], [39] at cell junctions, we infer that normal Merlin function is dependent on its ability to localize, at least transiently, to both the apical membrane and the junctional region, and that Merlin might normally traffic between these membrane domains.
